# Macular Carotenoid Supplementation Improves Visual Performance, Sleep Quality, and Adverse Physical Symptoms in Those with High Screen Time Exposure

**DOI:** 10.3390/foods6070047

**Published:** 2017-06-29

**Authors:** James M. Stringham, Nicole T. Stringham, Kevin J. O’Brien

**Affiliations:** 1 Nutritional Neuroscience Laboratory, Department of Psychology, University of Georgia, Athens, GA 30602, USA; 2 Interdisciplinary Neuroscience Program, Biomedical and Health Sciences Institute, University of Georgia, Athens, GA 30602, USA; ntstringham@gmail.com; 3 Vision Sciences Laboratory, Department of Psychology, University of Georgia, Athens, GA 30602, USA; Obrien.psych@gmail.com

**Keywords:** lutein, zeaxanthin, mesozeaxanthin, macular pigment, screen time, visual performance, sleep quality, computer vision syndrome

## Abstract

The dramatic rise in the use of smartphones, tablets, and laptop computers over the past decade has raised concerns about potentially deleterious health effects of increased “screen time” (ST) and associated short-wavelength (blue) light exposure. We determined baseline associations and effects of 6 months’ supplementation with the macular carotenoids (MC) lutein, zeaxanthin, and mesozeaxanthin on the blue-absorbing macular pigment (MP) and measures of sleep quality, visual performance, and physical indicators of excessive ST. Forty-eight healthy young adults with at least 6 h of daily near-field ST exposure participated in this placebo-controlled trial. Visual performance measures included contrast sensitivity, critical flicker fusion, disability glare, and photostress recovery. Physical indicators of excessive screen time and sleep quality were assessed via questionnaire. MP optical density (MPOD) was assessed via heterochromatic flicker photometry. At baseline, MPOD was correlated significantly with all visual performance measures (*p* < 0.05 for all). MC supplementation (24 mg daily) yielded significant improvement in MPOD, overall sleep quality, headache frequency, eye strain, eye fatigue, and all visual performance measures, versus placebo (*p* < 0.05 for all). Increased MPOD significantly improves visual performance and, in turn, improves several undesirable physical outcomes associated with excessive ST. The improvement in sleep quality was not directly related to increases in MPOD, and may be due to systemic reduction in oxidative stress and inflammation.

## 1. Introduction

Prior to the advent of artificial lighting, the sun was the primary source of light. When the sun sets today, however, one is hard-pressed to find darkness—illumination from light-emitting diodes (LEDs), and incandescent, fluorescent, and xenon-arc sources (among others) provide indoor and outdoor illumination. The effects of exposure to this seemingly unnatural technological adaptation are starting to become evident, with several studies indicating undesirable associations between exposure to artificial light at night and both reduced sleep quality [1,2] and diminished alertness during the day [3]. A relatively recent concern is the excessive near-field exposure to handheld and other electronic light-emitting devices. Estimates from large population surveys in developed countries indicate that 35% of people born between the years of 1965 and 1996 spend at least 9 h/day on digital devices, such as smartphones, tablets, or computers [4], and a recent report indicates that the average American spends 10 h and 39 min viewing screens [5]. Such intense use of these devices has been found to have undesirable effects not only on sleep quality and alertness during waking hours, but also on parameters of physical health (e.g., neck and eye strain, eye fatigue, headache; [6,7,8]) and cognitive performance (e.g., poor inhibitory control; [9]). The prevalence of complaints associated with excessive use of computers and other digital devices is so great that the common ocular and physical effects have been collectively termed “Computer Vision Syndrome” (CVS; for a review please see [10]). An additional concern is the potential for retinal damage from cumulative, excessive exposure to the relatively high-energy, short-wavelength (blue) light produced by these devices. In the case of LED-based devices, for example, a vivid white background is composed of blue light (emitted from an electrically activated phosphor) that is either passed through a yellow filter, or mixed with the output of red and green LEDs [11]. In either case, blue is the peak of the overall output of the LED. Although the blue component in an LED is relatively strong, it is not nearly intense enough to produce acute damage in the retina. There is, however, potential for long-term, cumulative effects, including cellular damage arising primarily from increased oxidative stress [12]. Because of their consistency, efficiency, and durability, LEDs have become the prevalent light source for devices ranging from smartphones to computer displays to televisions [4].

The potential for damage from blue light in the retina has been met with a favorable adaptation in the eye: a diet-derived, blue-absorbing pigment composed of the dietary carotenoids lutein (L) and zeaxanthin (Z). L and Z are found in relatively high concentrations in leafy-green vegetables [13], and along with the Z isomer mesozeaxanthin (MZ), are deposited in rich concentrations in the macular retina [14]. Due to their specific location, they are referred to as the macular carotenoids (MC); due to their yellow–orange pigmentation, they are collectively known as macular pigment (MP). All three MCs are potent antioxidants, which forms the basis of their putative role in protecting the retina from cumulative damage that can result in age-related macular degeneration (AMD) [15,16]. Indeed, the fovea’s metabolic demand for oxygen, and resulting potential for oxidative stress, is the highest of any tissue in the human body [17]. Because of its yellow–orange pigmentation and its location anterior to the photoreceptors, MP also acts as a blue light filter that protects against photo-oxidation of the vulnerable lipid-rich photoreceptor outer segments. Additionally, MP has been shown previously to significantly improve several aspects of visual performance that may be related to outcomes associated with excessive ST exposure, such as contrast sensitivity [18,19,20,21], temporal vision [22,23,24], and several aspects of visual performance in glare, including visual discomfort [25,26], disability glare [27,28], and photostress recovery [27,28,29].

The present study was motivated not only by the potential deleterious effects of the precipitous recent increase in the use of near-field technological devices (e.g., smartphones), but also the fact that the optical density (OD) of MP (MPOD) is (given its source in the diet) notoriously variable among people, ranging from 0 to well over 1 logarithmic unit of OD [30]. If there exists a benefit for MPOD in terms of negative effects of excessive ST, are those with little or no MP compromised? This question was addressed by the two primary goals of our study: (1) to characterize the baseline relationships between MPOD, visual performance measures, sleep quality, and physical indicators of excessive screen time (ST) in young, healthy adults; and (2) to determine effects of a 6-month MC supplementation on these same outcomes.

## 2. Materials and Methods

This study was reviewed and approved by the University of Georgia Institutional Review Board. Informed consent was obtained for each subject, and the study adhered to the tenets of the Declaration of Helsinki. Forty-eight subjects, recruited from the population of students at the University of Georgia in Athens, Georgia, participated in this 6-month, double-blind, randomized, placebo-controlled supplementation trial. Subjects were healthy, college-aged (18–25, mean = 21.2 years; 25 female/23 male) non-smokers with a body mass index (BMI) <27. The concern with BMI involves not only good general health, but also the possibility that, being lipid-soluble, supplemented macular carotenoids may be deposited in adipose tissue, thus reducing the fraction deposited in the retina. A BMI of 27 is higher than “normal” (18.5–24.9) but falls well below “obese” (BMI = 30 or greater). We had seven participants who approached 27, and five of them were athletes (whose muscle structure accounted for the relatively high BMI). Subjects were instructed to maintain their current diet; those that were planning on changing their diet (for whatever reason) were excluded from consideration for the trial. For those subjects enrolled in the trial, stability of diet was evaluated via questionnaire at each visit. In consideration of vision testing, all subjects had uncorrected or contact lens-corrected visual acuity of 20/20 or better in the test (right) eye, and had no current or previous history of ocular pathology. Subjects wearing spectacles for vision correction were not considered for the study, due primarily to potential reflection and coating-mediated light absorption issues with glare testing. Subjects were also screened to ensure that they spent at least 6 h daily viewing screens at a distance of 3 feet or less. Items specific to smartphones, tablets, computers, and television viewing were included on the questionnaire. Measures of all visual and physical parameters (detailed below) were taken at baseline, 3 months and 6 months.

### 2.1. Macular Carotenoid Supplementation

Subjects were randomly assigned to either placebo (*n* = 13) or MC supplement (*n* = 35) groups. Pills for each group were identical, brown-colored, soft gelatin capsules. Placebos contained no L, Z, or MZ, but only safflower oil. Independent analysis indicated that the active supplement pills (Lutemax 2020 formulation) contained 24 mg of L, Z, and MZ suspended in safflower oil at a ratio of 83%:10%:7%, respectively. All reported values were within +/−5% sum total variability. A previous investigation [31] of these supplements demonstrated good bioavailability, as indicated by significant serum response. Subjects were instructed to ingest one pill with a meal (preferably lunch or dinner) every day. Compliance was ensured with weekly phone calls and pill counts.

### 2.2. Measurement of Macular Pigment Optical Density (MPOD) 

MPOD was assessed with a non-invasive, perceptual task called heterochromatic flicker photometry (HFP). A densitometer (Macular Metrics Corp., Rehoboth, MA, USA) described by Wooten et al. [32] was used for this purpose. The densitometer, detailed measurement procedures, and the principle of HFP have been fully described in earlier publications (e.g., [33,34]). Briefly, subjects are presented with two superimposed lights that are temporally alternated in square-wave counterphase. This creates the perception of a flickering disc of light for the subject. The peak (550 nm) of the spectral composition of one of the lights is chosen to bypass the absorption of MP, and the other (460 nm) is strongly absorbed by MP. The subject’s task is to adjust the relative radiance of the two lights until a percept of no flicker, due to perceived isoluminance, is achieved. All other factors being equal, a subject that requires more short-wave (i.e., 460 nm) relative to middle-wave (i.e., 550 nm) light to achieve null flicker has higher MPOD. This task is performed for the locations of interest within the fovea, which presumably contain MP, and for a reference location in the parafovea that does not (about 7° eccentricity). To obtain a measure of MPOD at a given test locus, the logarithmic ratio of short- to middle-wave radiance (for null flicker) at the reference location is subtracted from the corresponding logarithmic ratio found at the test locus. We obtained spatial profiles of MPOD at each visit, with measures at 10’, 20’, 30’, 1.75 degrees, and 2.75 degrees of retinal eccentricity. MPOD for centrally-viewed relatively small (less than or equal to 1.5 degrees) circular targets has been shown to correspond to the edge of the disc [35]. Therefore, our stimuli of 20’, 40’, and 10 of visual angle corresponded to 10’, 20’, and 30’ retinal eccentricities. For measures corresponding to 1.75 and 2.75 degrees of retinal eccentricity, subjects viewed a small fixation dot in the center of annuli with diameters of 3.5 and 5.5 degrees, respectively. Due to the nature of the variables tested in the study (involving relatively large viewing angles), we averaged MPOD across participants’ spatial distributions. Although this technique effectively compresses any absolute increases in OD, it yields a more reasonable estimate of the overall effect of MP across the macula. The total time spent on MPOD measurement was 15 min per session.

### 2.3. Measurement of Temporal Vision (CFF)

Subjects were presented with a 550 nm narrow-band (10 nm at half peak), 1-degree disc of light that was alternated on and off in square-wave counterphase. Thresholds for flicker fusion (when the light was flickering at a rate such that there was no longer any flickering or pulsing evident) were obtained for both ascending and descending trials. For ascending trials, the rate of flicker was set well below the critical flicker fusion frequency (CFF) threshold, and at a rate of roughly 1 Hz/second, the flicker rate was increased by the experimenter until the participant indicated complete flicker fusion. On descending trials, the rate of flicker was set well above the CFF threshold, and then decreased roughly 1 Hz/second until the participant just noticed minimal pulsing or flicker. The overall threshold for the CFF task was calculated as the average of two ascending and two descending thresholds; the task took 1 min for subjects to complete.

### 2.4. Physical Indicators of Excessive Screen Time

During each visit, participants completed a short questionnaire regarding the weekly frequency of occurrence of five outcome variables typically associated with excessive ST: headache, blurry vision, neck strain, eye strain, and eye fatigue. If a specific item occurred less frequently than once/week, but still occurred on a monthly basis (e.g., twice/month), participants were instructed to write down the monthly frequency next to the item. For calculation purposes, a month was considered four weeks; monthly frequency was therefore simply divided by four to yield a weekly value. The questionnaire took approximately 5 min to complete.

### 2.5. Sleep Quality

Sleep quality was evaluated with the Pittsburgh Sleep Quality index (PSQI; [36]), a 19-item self-rated questionnaire. The PSQI determines the number of undesirable sleep symptoms (e.g., “wake up in the middle of the night”), which are then weighted for severity and used to calculate the overall sleep quality score. The questionnaire took subjects approximately 5 min to complete.

### 2.6. Contrast Sensitivity Testing

Contrast sensitivity (CS) testing was conducted on the same computer/monitor as described above. A subject’s threshold for detection of a Gabor patch’s orientation (tilted right or left 45 degrees from vertical) was determined for a single stimulus, a 6 cycle/degree target subtending 2 degrees of visual angle. A two-alternative, forced-choice staircase procedure was implemented to determine a subject’s contrast threshold. If there was no response, it was recorded as incorrect. Contrast was specified as Michelson contrast:

$$\frac{Lmax-Lmin}{Lmax+Lmin}$$

where *Lmax* and *Lmin* represent the maximum and minimum luminance in a grating, respectively. Twenty-five stimulus presentations were used to determine a threshold, and trials always started with the Gabor set to maximum contrast (90% Michelson contrast). On correct responses, the contrast of the Gabor was decreased by 27% of its previous value. Incorrect responses resulted in an increase of 21% of the previous Gabor’s contrast value. Based on the results of an ideal observer model, these values most accurately predicted actual contrast thresholds for a trial consisting of 25 stimulus presentations, averaging the last three reversals. The subjects typically produced five or more reversals; actual thresholds were determined by computing the average of the last three reversals. Two thresholds were determined at each visit; the average of the two thresholds was taken as the true threshold, and used for statistical analysis. A 1-min rest period was allowed between each trial. Total time spent on contrast sensitivity testing was roughly 10 min.

### 2.7. Disability Glare/Photostress Recovery

A custom apparatus for assessing disability glare (DG) and photostress recovery (PSR) time was created to provide sufficient intensity of light and a uniform spatial distribution with minimal reliance on optics. A microcontroller, coupled to high-intensity cool-white LEDs, provided a pulse-width modulated signal, which permitted the glare and photostress sources to be held at an adjustable intensity for any needed duration, while maintaining a high degree of linearity and stability in adjustment. The LEDs appear white by virtue of the phosphor emitting a strong blue component and a broad yellow–orange component. For human vision, a mix of blue and yellow sources will result in a range of whites, denoted “cool” to “warm” (e.g., [37]). For the DG source, a ring of 47 LEDs were placed on a circuit board with a viewing hole in its center. The LEDs were arranged in a circular pattern with a 20 mm radius to create a plane of uniform illumination at the pupil when held approximately 25 mm from the subject’s pupil. The photostress source was created by using 45 of the same high-intensity cool-white LEDs arranged in an approximately square pattern with 5 mm center-to-center spacing of the LEDs. This arrangement was used to create a uniform, circular illumination pattern on an acrylic diffuser that subtended 10 degrees of visual angle and maintained an illumination of 12,000 lux. The driving microcontroller was given commands over a serial connection to coordinate the intensity of the glare and photostress sources with a custom-made program that also produced visual targets for the subject.

To control for any possible interocular effects (however unlikely), only the right eye was used for all vision testing. For both DG and PSR experiments, the target was a 6 cycle/degree, 15% Michelson contrast Gabor patch that subtended 2 degrees of visual angle. The Gabor patch was presented centrally on an LCD monitor. The background luminance was 20 cd/m^2^, and appeared medium-gray to subjects. For the PSR experiment, subjects were aligned to the optical system via an adjustable chin and forehead rest. Once comfortable, subjects were instructed to direct their gaze at the center of the monitor. The photostress light (12,000 lux) was then presented for 8 s. Subjects were asked to look directly at the light, and try to refrain from blinking or closing their eyes. After the exposure was complete, subjects were advised to blink and look in the center of the monitor for the Gabor target, which was tilted 45 degrees left or right from vertical. Once the target was detected, subjects pressed either the left or right arrow key on a keyboard to indicate which direction the target was leaning. If correct, subjects were again presented with the photostress light for 8 s. This procedure was repeated until a total of 5 recovery thresholds were obtained. 

For DG testing, subjects viewed the Gabor patch target through the ring of LEDs. To guard against visual adaptation effects or potential subject bias, the Gabor patch was made to vertically tilt back and forth 45 degrees every second. The experimenter adjusted the intensity of the LED ring to the point at which the veiling glare was sufficient to prevent a subject from detecting the tilting of the Gabor patch. Four thresholds were obtained: two approaching from below, and two approaching from above the visibility threshold. The four thresholds were averaged to form the overall DG threshold. Total time per session for these tasks was roughly 15 min.

### 2.8. Statistical Analysis

The statistical and graphing program OriginPro 9.3 (Northampton, MA, USA) was used to conduct repeated-measures ANOVA, Pearson product–moment correlations, and generate figures for the manuscript. Levene’s test for homogeneity of variance was used and determined to be non-significant. In consideration of the unequal N for the two groups, a power analysis was conducted to determine adequate sample sizes in order to detect effects of supplementation on all of the outcome measures. The statistical power rate 1−β was set at 0.80; a 20% change in visual performance or physical indicator status in treatment groups, a standard deviation of 20%, and α = 0.05 were parameters used for calculation. With the placebo group set at *n* = 12, the power calculation determined that the treatment group required 30 subjects to detect effects (if present). We recruited additional subjects for the study, assuming some attrition.

## 3. Results

### 3.1. Baseline Results

At baseline, several measures were found to be significantly correlated. For the visual measures, MPOD was correlated with PSR (*r* = −0.48; *p* < 0.001), DG (*r* = 0.40; *p* = 0.03), CFF (*r* = 0.31; *p* = 0.035), and CS (*r* = 0.29; *p* = 0.048). CFF and CS were also significantly correlated (*r* = 0.41; *p* = 0.0036), as were DG and PSR (*r* = −0.32; *p* = 0.04). Lastly, a marginally significant correlation was found for PSR and CFF (*r* = 0.30; *p* = 0.06). One significant correlation was determined between visual measures and physical indicators of excessive ST: PSR and eye fatigue (*r* = 0.35; *p* = 0.04). Several marginal correlations were determined at baseline for the measures of physical indicators of excessive ST, including MPOD and eye fatigue (*r* = −0.26; *p* = 0.074), eye strain (*r* = −0.27; *p* = 0.061), and headache frequency (*r* = −0.25; *p* = 0.09). Additionally, there were marginal correlations found between headache frequency and both eye strain (*r* = 0.28; *p* = 0.068) and neck strain (*r* = 0.24; *p* = 0.088); frequency of neck strain and eye strain were also marginally correlated (*r* = 0.26; *p* = 0.079). Table 1 presents means and standard deviations for every variable assessed in the study, for both placebo and treatment groups, at each measurement time point. Average near-field ST was determined to be 7.83 +/− 0.48 h for our sample, with no difference between placebo (8.06 +/− 0.61) or treatment (7.75 +/− 0.41) groups.

### 3.2. Effects of Supplementation

**Figure 1:**
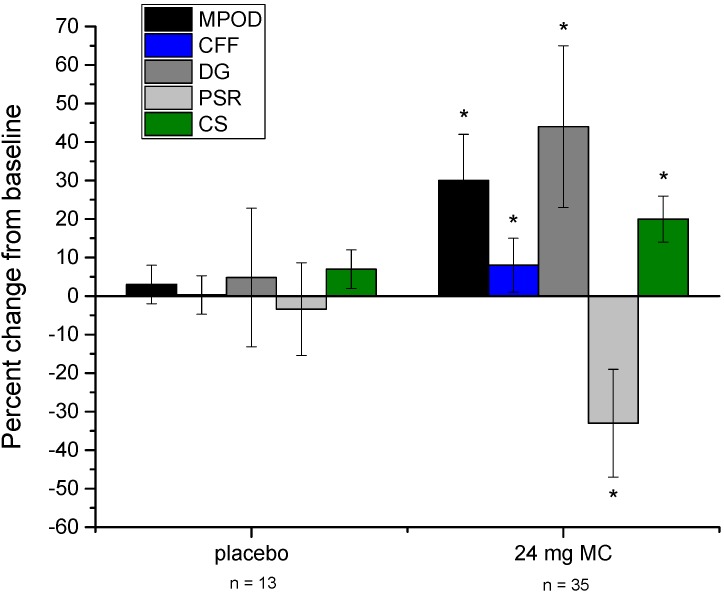
Percent change from baseline for MPOD and visual performance measures (noted in legend), at 6 months for both placebo and treatment groups. Error bars are +/− 1 SD. Asterisks denote statistically significant difference from placebo (*p* < 0.05). Definitions: MPOD = macular pigment optical density; CFF = critical flicker fusion frequency; CS = contrast sensitivity (plotted in terms of relative percentage change, not absolute sensitivity change); DG = disability glare; PSR = photostress recovery.

**Figure 2:**
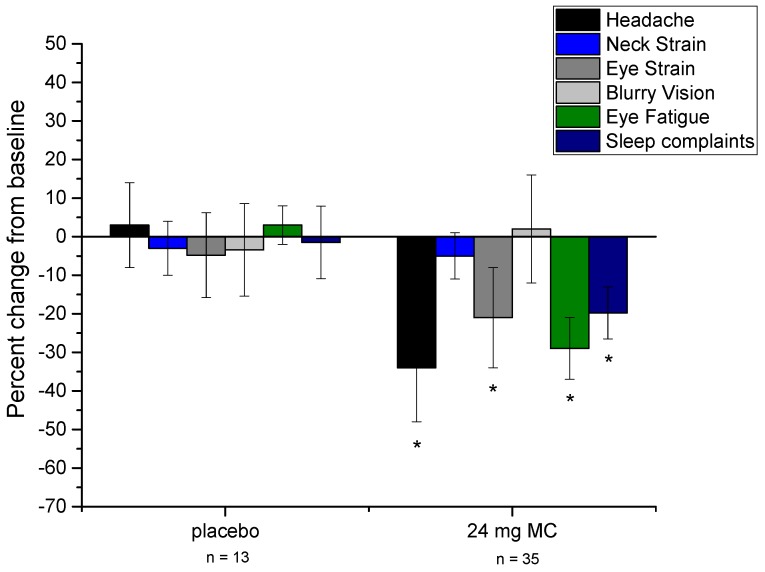
Percent change from baseline for physical indicators of excessive screen time (ST) (including sleep quality), at 6 months for both placebo and treatment groups. Error bars are +/− 1 SD. Asterisks denote statistically significant difference from placebo (*p* < 0.05).

Compared to the placebo group, those in the MC supplementation group changed significantly for several of the variables measured in the study. See Figure 1 for a graphical representation of changes in visual performance variables throughout the study. To clarify (as with the other variables presented in Figure 1), CS data are plotted as the percent change from baseline, not absolute change in CS (raw summary data are presented in Table 1). Figure 2 presents physical indicator variables in the same fashion. Repeated-measures ANOVA revealed that, versus placebo, CFF (*p* = 0.023) and overall sleep quality (*p* = 0.025) changed significantly after 3 months of supplementation. At 6 months, several additional variables were found to have changed significantly versus placebo. These included MPOD (*p* = 0.015), CFF (*p* < 0.001), sleep quality (*p* = 0.01), CS (*p* = 0.002), DG (*p* = 0.021), PSR (*p* = 0.011), headache frequency (*p* = 0.029), eye strain (*p* = 0.046), and eye fatigue (*p* = 0.016). Frequency of blurry vision and neck strain were not found to change appreciably throughout the study in either the placebo or treatment group. Subjects’ consumption of foods that contain L and Z did not change throughout the study period (*p* = 0.53), and neither did their estimates of ST (*p* = 0.62).

## 4. Discussion

There are two major points to take away from the results of this study. First, baseline data suggest that MPOD is associated with several visual and physical benefits for those who view near-field screens for at least 6 h daily. At baseline, MPOD was significantly related to each of the visual performance measures, and marginally related to frequency of headaches, eye strain, and eye fatigue. Second, 6 months of supplementation with 24 mg of MCs led to significant improvements in each of the visual measures (including a significant increase in MPOD), a significant improvement in sleep quality, and a significant reduction in the frequency of headaches, eye strain, and eye fatigue (see Figure 1 and Figure 2).

Given the rich deposition of the MCs in the foveal retina, the most parsimonious explanation of the effects determined in our study would seem to involve the deposition of the MCs in the retina as MP. Certainly this is the most plausible explanation for the visual performance results—baseline relationships and changes with MPOD augmentation for these measures have also been shown in previous studies (see e.g., [22,27,29]). Indeed, due to its blue light filtering capability, MPOD should have an appreciable effect on any visual task that involves foveating a target that contains blue light. Because the LEDs used for the DG and PSR tasks in the present study emitted a strong blue component, results of the glare portion of the study support this idea. Other results are less straightforward. For example, results from the CFF and CS tasks appear to be accounted for by different, perhaps neurophysiological mechanisms. The idea that increased MPOD can lead to increased “neural efficiency” has been proposed to explain the significantly higher CFF seen in those with higher MPOD [23]. In this scenario, the presence of MCs (such as L) throughout the visual system would increase efficiency of signaling among visual neurons that serve temporal vision. Alternatively, it may be that the increased CFF seen with higher MPOD is an adaptation of the visual system to offset the absorption of short-wavelength light in order to maintain uniform color vision across the visual field [24]. In terms of the CS results, light filtration cannot account for an increase in CS because the percent absorption of the light vs. dark bars of the grating is equivalent. Moreover, an overall reduction in luminance would serve to decrease signal-to-noise ratio, which runs counter to both our baseline and supplementation results. The mechanism must, therefore, involve something other than modification of the retinal image by MPOD. One possibility is enhancement of the neurophysiology of the retina, where increased MPOD, via antioxidant action, would serve to increase the metabolic efficiency of the visual cycle [18,19]. This could lead to a favorable redox status and a corresponding increase in the signal-to-noise ratio in the output of center-surround visual receptive fields [38], which would ultimately lead to increased CS.

The results of the physical indicators of excessive ST portion of the study are somewhat unexpected. MPOD is (by definition) located in the eye, and by extension expected to have its effects on vision. Marginal associations and significant improvements were seen, however, in a few of the physical indicator variables. The question that arises from this seeming discrepancy involves the link between the two—how can something with such a specific location have its effects elsewhere in the body? With regard to the significant improvement in eye fatigue with supplementation, a plausible explanation could involve the aforementioned increased efficiency of the visual cycle seen with increased MPOD. Although light from smartphone, tablet or computer screens is not typically very intense, there is nevertheless prodigious demand for metabolic resources when viewing any light source continually. If the visual system is able to regenerate photopigment more efficiently, it may keep the state of visual adaptation at a level that serves to increase visual sensitivity, which may manifest as an increase in CS (which is what was found in the present study; see also studies [18,19], and improved ratings of visual fatigue). Alternatively, perhaps there is a mild discomfort experienced by those with relatively low MPOD that may result in mild squinting of the eyes. Squinting of the eyes is a common sign of visual discomfort, and is usually associated with lights that are exceedingly bright, and is significantly worse for short-wavelength (blue) lights [25]. Perhaps the emission of low-level blue light from near-field devices is sufficient to produce a very low-level squinting of the eyes? If this low-level squinting were to persist long-term (e.g., for several hours), it may manifest as visual fatigue or eye strain, and could explain our baseline and MC supplementation results. Following this line of reasoning, the reduction in headache frequency (which is also related to long-term eye squinting [39]) may arise from reduced long-term squinting of the eyes. The apparent discrepancy between MP’s location and the seemingly peripheral physical indicator effects may be reconciled by the several ways in which MP serves visual performance, including reduced squinting in blue light.

In terms of sleep quality, there are a couple of plausible explanations for the improvements that are seen in the MC supplement group. From an ocular standpoint, the body’s circadian “clock” is significantly refined by input from the blue-sensitive melanopsin system [40]. This non-visual photopigment, when stimulated by short-wavelength radiation (e.g., from the sun or from digital devices), signals, via the suprachiasmatic nucleus, to reduce secretion of melatonin from the pineal gland, and produce wakefulness. It would seem, therefore, that an increase in MPOD (which absorbs short-wavelength light) would lead to reduced stimulation of the melanopsin system and consequently less trouble getting to sleep/perhaps better quality sleep. MP and melanopsin, however, occupy distinctly different areas of the retina—MP (as noted above) is richly deposited in the foveal region whereas melanopsin is found widely dispersed extrafoveally [41]. Increases in MPOD would therefore not be expected to exert an influence on absorption of blue light by melanopsin. Indeed, we determined no baseline or commensurate change relationship with supplementation between MPOD and sleep quality. This suggests that the positive effects of MC supplementation on sleep quality are perhaps systemic, and that MPOD is simply a covariate in this case. In other words, supplementation with MCs have the effect of increasing MPOD, but also have wide-ranging systemic antioxidant [42] and anti-inflammatory [43] effects. It could be, therefore, that systemic reduction of oxidation and inflammation ultimately manifests as better overall sleep quality. Additionally, a recent report suggests that supplementation with MCs for six months significantly reduced psychological stress and serum cortisol [44]. Because psychological stress is often noted as a significant factor for insomnia [45], its reduction may account for the beneficial effect on sleep quality seen in the present study.

## 5. Conclusions

The significant visual and physical issues associated with the relatively recent, precipitous increase in the use of devices that necessitate near-field viewing of screens is reason for concern. The results of the present study speak to several of these issues, and offer a benign, nutrition-based therapy for reducing the incidence of many undesirable outcomes associated with excessive ST. Although the effects presented in this study appear to be reasonably uniform among subjects and generally commensurate with retinal response to MC supplementation, they motivate the need for additional, larger studies in research in populations other than college-aged individuals. For example, in those who are in the age group of 40–45 years, pre-clinical presbyopia may exacerbate issues associated with excessive ST. Without the aid of proper optical magnification, those with diminished accommodative ability are often seen to squint in order to visually resolve near material. As noted above, it may be that long-term squinting (in this case to promote focus) is the root of several of the negative outcomes of excessive ST. The effects of ST on children are just starting to be appreciated, and they represent another population of interest. Accommodative ability is typically not an issue in children, but there may be other, less obvious issues and effects in this population. For example, children younger than 10 years old are known to have exceptionally clear crystalline lenses [46], and may have not had sufficient time/dietary habits to accumulate appreciable MP. The clarity of the lens (which normally yellows slightly with age) means that more high-energy blue light will reach the retina, where a lack of MP may compromise the ability of the retina to deal with the oxidative stress. Given the recent dramatic rise in the use of handheld devices/computers by this age group [4], it would be interesting to determine if a nutritional intervention could help with potential visual, physical, or perhaps behavioral outcomes.

## Figures and Tables

**Table 1:** Raw summary data (means and standard deviations (SD)) for all variables, for both groups, at all study time points. Asterisks denote statistically significant difference from placebo (*p* ≤ 0.05). Definitions: MPOD = macular pigment optical density; CFF = critical flicker fusion frequency; CS = contrast sensitivity (absolute threshold percent sensitivity); DG = disability glare; PSR = photostress recovery.

Variable	Placebo Group (*n* = 13)						Macular Carotenoid Group (*n* = 35)					
	Baseline	SD	3 months	SD	6 months	SD	Baseline	SD	3 months	SD	6 months	SD
MPOD	0.372	0.119	0.368	0.135	0.383	0.132	0.376	0.133	0.421	0.151	0.472*	0.144
Total Number of Undesirable Sleep Symptoms	5.46	2.06	5.69	2.69	5.38	2.02	4.74	2.11	3.89*	1.55	3.8*	1.51
CFF (Hz)	25.23	1.8	25.33	1.74	25.38	1.84	25.05	2.13	26.31*	1.69	27.12*	1.96
CS threshold (percent contrast)	4.25	0.38	4.21	0.33	4.01	0.31	4.63	0.46	4.13	0.39	3.73*	0.34
Headache Frequency (weekly)	2.65	1.21	2.57	1.16	2.73	1.19	2.78	1.12	2.02	0.94	1.83*	0.91
Eye strain (weekly)	2.11	2.61	1.96	2.54	2.01	2.72	2.4	2.42	2.04	2.11	1.71*	1.82
Eye Fatigue (weekly)	2.88	2.43	2.91	2.75	2.96	2.62	3.03	2.77	2.61	2.64	2.18*	2.23
Blurry vision (weekly)	1.33	2.46	1.37	2.55	1.29	2.29	1.43	2.54	1.47	2.48	1.46	2.59
Neck strain (weekly)	3.36	3.81	3.41	3.77	3.27	3.69	3.55	3.9	3.53	3.74	3.38	3.49
DG (nominal light output)	101.78	29.15	105.44	31.25	103.87	33.35	81.67	23.85	106.31	29.83	117.66*	35.42
PSR (seconds)	10.54	3.41	10.79	4.02	10.11	3.61	11.03	3.99	8.89	3.41	7.41*	2.91
